# Computed Tomographic Study in 73 Pet Rabbits (*Oryctolagus cuniculus*): Evaluation of the Correlation Between Dental and Thoracic Pathology

**DOI:** 10.3390/ani16020342

**Published:** 2026-01-22

**Authors:** Glenda Murciano, Iván Alonso-Fernández, Rosa Novellas, Osvaldo Fonseca-Rodríguez, Jaume Martorell

**Affiliations:** 1 Hospital Clínic Veterinari, Universitat Autònoma de Barcelona, 08193 Cerdanyola del Vallès, Barcelona, Spain; ivan.alonso@uab.cat (I.A.); rosa.novellas@uab.cat (R.N.); jaumemiquel.martorell@uab.cat (J.M.); 2 Departament de Medicina i Cirurgia Animals, Facultat de Veterinària, Universitat Autònoma de Barcelona, 08193 Cerdanyola del Vallès, Barcelona, Spain; 3 Institut de Recerca i Tecnologia Agroalimentàries (IRTA), Programa de Sanitat Animal, Centre de Recerca en Sanitat Animal (CReSA), Campus de la Universitat Autònoma de Barcelona (UAB), 08193 Cerdanyola del Vallès, Barcelona, Spain; osvaldo.fonseca@irta.cat; 4 Unitat Mixta d’Investigació IRTA-UAB en Sanitat Animal, Centre de Recerca en Sanitat Animal (CReSA), Campus de la Universitat Autònoma de Barcelona (UAB), 08193 Cerdanyola del Vallès, Barcelona, Spain

**Keywords:** *Oryctolagus cuniculus*, dental disease, odontogenic infection, oral–lung axis, thoracic pathology, computed tomography

## Abstract

**Background:** Dental and respiratory diseases are common in companion rabbits. While upper respiratory tract disorders have been described in association with dental pathology, the possible correlation between dental disease and pulmonary lesions has not yet been investigated. **Methods:** A cross-sectional study was conducted on client-owned rabbits presented for suspected dental disease that underwent head and thoracic computed tomography (2012–2025) at a veterinary teaching hospital. Seventy-three rabbits met the inclusion criteria (mean age 4.96 years). **Results:** Dental involvement was present in 81% (59/73) of the rabbits and pulmonary lesions in 29% (21/73). Among rabbits with dental disease, frequent osseous findings included osteomyelitis (49%), inflammatory resorption (42%), and maxillary deformities (33%). Pulmonary abnormalities occurred in 32% of rabbits with dental disease compared to 14% of those without. In Poisson models with log link adjusted for age and sex, dental disease was not significantly associated with pulmonary lesions (prevalence ratio [PR] 2.14; 95% CI 0.55–8.38; *p* = 0.275). Sensitivity analyses using 1:1 matching with cluster-robust variance (PR 3.24; 95% CI 0.65–16.1; *p* = 0.152) and overlap-weighted doubly robust estimation (PR 2.08; 95% CI 0.42–10.4; *p* = 0.371) yielded directionally positive but imprecise results. **Conclusions:** Computed tomography proved valuable for the evaluation of thoracic comorbidities in rabbits with dental disease. Although dental and pulmonary abnormalities were often observed together, a statistically significant association was not established. Larger, prospective studies are warranted to further clarify this relationship.

## Simple Summary

Dental and respiratory diseases are common in pet rabbits and may occur together because of the close anatomical relationship between the teeth, sinuses, and airways. This study analyzed computed tomography scans from 73 rabbits examined for dental disease to investigate whether lung changes were also present. Most rabbits with dental disease showed bone and soft-tissue alterations involving the peripheral tissues, confirming that oral pathology can extend to nearby structures. Although pulmonary abnormalities were observed more frequently in rabbits with dental disease than in those without, this difference was not statistically significant. Computed tomography proved to be an extremely valuable tool for identifying changes in both dental and thoracic structures. These findings highlight the importance of comprehensive imaging in rabbits with chronic dental conditions and encourage further studies to clarify possible systemic effects of oral disease.

## 1. Introduction

In recent years, companion rabbits (*Oryctolagus cuniculus*) have become increasingly popular as household pets, leading to a parallel rise in the demand for advanced veterinary care tailored to their unique anatomical and physiological needs [1]. Among the most frequently diagnosed health problems in pet rabbits are dental and respiratory diseases [2]. Dental disease in rabbits is particularly prevalent due to their continuously growing elodont dentition, which requires regular occlusal wear [3,4]. Malocclusion, elongated tooth roots, and associated abscessation are frequently encountered in clinical practice and are often exacerbated by dietary imbalances or congenital malformations [5,6,7].

Respiratory diseases, on the other hand, are also a significant concern in rabbits, often presenting as chronic or recurrent conditions that can be challenging to diagnose and manage [5]. Clinical manifestations may range from nasal discharge and sneezing to dyspnea and cyanosis [5,8]. A growing body of literature suggests that dental and respiratory diseases in rabbits may not be isolated phenomena but rather interconnected processes, particularly due to the anatomical proximity of the upper dental arcade to the nasal cavity, nasolacrimal ducts, and paranasal sinuses [8,9]. A recent study using computed tomographic evaluation enabled the characterization of nasal and paranasal disease in rabbits and highlighted the association between rhinitis, sinusitis, and dental disease, although the correlation with pulmonary abnormalities was not statistically analyzed [10].

Advanced imaging modalities, particularly computed tomography (CT), have revolutionized the diagnostic capabilities in exotic animal medicine [11]. CT offers high-resolution, cross-sectional images that allow superior visualization of the dental structures and the soft tissues, including the pulmonary parenchyma and airways, without the superimposition inherent in conventional radiography [11]. In the context of rabbit medicine, CT has proven to be a valuable tool for identifying signs of dental disease, such as periapical lucency, alveolar bone lysis, and tooth root elongation, as well as for detecting subtle pulmonary changes that may be overlooked in thoracic radiographs [11,12]. While computed tomography has emerged as a gold standard for diagnosing dental pathology in rabbits due to its ability to visualize three-dimensional structural abnormalities [11], the potential interplay between dental disease and pulmonary pathology remains underexplored in this species. Recent studies in companion animals and humans highlight the aerodigestive axis as a critical interface between oral health and respiratory outcomes. In dogs, dysphagia, gastroesophageal reflux, and aspiration events secondary to dental or esophageal dysfunction are well-documented contributors to pneumonia [13,14]. Similarly, human studies demonstrate that oral pathogens and inflammatory mediators from periodontal disease can exacerbate chronic respiratory conditions, such as asthma, or trigger a lung disease such as aspiration pneumonitis [15,16,17,18]. These findings suggest that dental pathology may act as a nidus for respiratory disease through direct mechanical effects, microbial translocation, or systemic inflammation. In rabbits, however, the anatomical and physiological context differs from that of humans and dogs. Rabbits are obligate nasal breathers, and in the normal anatomical position the epiglottis lies dorsal to the soft palate, efficiently separating the oral cavity from the trachea and limiting oral airflow and direct tracheal access via the mouth [5]. This configuration markedly reduces the likelihood of primary oral aspiration as a mechanism for pulmonary disease. Nevertheless, severe dental disease may impair mastication and swallowing efficiency, potentially predisposing affected rabbits to abnormal deglutition dynamics and secondary aspiration events, particularly under conditions of pain, stress, or altered consciousness. While these mechanisms remain less well defined than in non-obligate nasal-breathing species, they warrant further investigation using multimodal diagnostic approaches.

Despite these reasonable associations, there are no studies that have systematically investigated the co-occurrence of dental and pulmonary diseases in rabbits using advanced imaging techniques.

Given the increasing availability and utility of CT in veterinary practice, there is a compelling need to explore the diagnostic and prognostic implications of identifying concurrent dental and pulmonary pathology in rabbits. A thorough understanding of the correlation between these conditions could lead to improved clinical outcomes, more targeted therapeutic approaches, and a greater appreciation for the complex pathophysiology underlying multisystemic disease presentations in this species.

The potential link between dental disease and pulmonary pathology in rabbits may be multifactorial. Dental abscesses, particularly those involving the maxillary arcade, can extend into the nasal cavity or sinuses, leading to secondary rhinitis, which can descend into the lower respiratory tract if untreated. Furthermore, chronic inflammation and infection associated with dental disease may compromise systemic immune function or serve as a nidus for hematogenous spread of pathogens to the lungs. Alternatively, impaired mastication due to dental malocclusion could exacerbate the risk of bronchial aspiration of food particles, potentially contributing to secondary respiratory disease. The present study was prompted by necropsy findings in pet rabbits showing concurrent severe dental disease and pulmonary lesions, including embolic hematogenous pneumonia and catarrhal purulent bronchopneumonia, which raised the question of whether such associations could also be identified in vivo through computed tomography.

The aim of this research was to evaluate, through computed tomographic imaging, the coexistence and potential correlation between dental disease and pulmonary pathology in pet rabbits.

By assessing a group of pet rabbits that underwent CT for dental disease, we sought to document the prevalence of concurrent pulmonary abnormalities and explore whether specific dental findings are statistically associated with thoracic changes. We hypothesized that rabbits with dental disease would exhibit a higher incidence of pulmonary pathology compared to those without dental abnormalities.

## 2. Materials and Methods

### 2.1. Study Design and Population

This was a cross-sectional, single-center study. The electronic medical database of the “Hospital Clínic Veterinari UAB” (Veterinary Teaching Hospital) was reviewed to identify rabbits presented with a clinical suspicion of dental disease that underwent a CT study between 2012 and 2025. Inclusion criteria consisted of adequate diagnostic image quality and the inclusion of both the head and thoracic regions in pre- and post-contrast series. The only exclusion criterion was the presence of significant intrathoracic diseases that prevented adequate expansion and assessment of the lung parenchyma. The decision for subject inclusion was made by a resident in small mammal medicine and surgery (G.M.) and a diagnostic imaging intern (I.A.), after agreement with a European College of Veterinary Diagnostic Imaging board-certified veterinary radiologist (R.N.) and a European College of Zoological Medicine (small mammal medicine and surgery specialist) board-certified veterinarian (J.M.). The study protocol and the use of patient data were approved by the veterinary teaching hospital clinical committee.

### 2.2. Imaging and Anesthesiology Procedures

All rabbits were initially sedated with a combination of alphaxalone (2 mg/kg IM; Alfaxan^®^ Multidose, Zoetis, Parsippany, NJ, USA), butorphanol (0.5 mg/kg SC; Torbugesic Vet, Zoetis, Parsippany, NJ, USA), and midazolam (0.5 mg/kg SC; Midazolam Normon, Normon, Tres Cantos, Madrid, Spain) to minimize the stress and facilitate the manipulation of the patients. Then, an intravenous catheter 24G (Introcan Certo, B. BRAUN, Melsungen, Germany) was placed either in the cephalic vein, the auricular marginal vein or the saphenous vein, based on the patient’s conformation and according to the operator’s technical preference. Additionally, if it was necessary, a dose of alphaxalone (0.5–1 mg/kg IV) was administered slowly until complete sedation was achieved.

CT examinations were acquired in soft tissue and bone algorithms, with the patient positioned in sternal recumbency, before and approximately 30 s after manual administration of iodinated contrast (2 mL/kg IV; XENETIX 300 mg/mL iodine, Guerbet, Villepinte, France). Scans were performed in a 16-slice helical CT scanner (General Electric Brivo CT 385, GE Healthcare, Chicago, IL, USA) with a slice thickness of 0.625 mm or 1.25 mm, an interslice interval of 0.625 mm or 1.25 mm, a collimation pitch of 0.5625:1, 120 kV, 50–90 mA and a matrix of 512 × 512. The field of view was specifically adjusted according to patient size.

### 2.3. Image Interpretation and Analysis

The images were retrieved from the Picture Archiving and Communication System (PACS) and transferred to a computer workstation (iMac 21-inch, Apple Inc., Cupertino, CA, USA). The studies were independently reviewed by two of the authors (I.A. and G.M.) using a medical DICOM image viewer software (OsiryX 13.0.1, 64 biy, Pixmeo, Bernex, Switzerland), by applying soft tissue (window width: 400 Hounsfield units, window level: 40 Hounsfield units) and bone (window width: 2500 Hounsfield units, window level: 480 Hounsfield units) windows. The results were supervised by a European College of Veterinary Diagnostic Imaging board-certified veterinary radiologist (R.N.), and the reviewers from the diagnostic imaging department (I.A.; R.N.) were completely blinded to the patients’ clinical data. CT images were initially analyzed in transversal sections, and multiplanar reconstructions in transverse, sagittal and dorsal sections were performed when considered necessary.

The head was assessed for the presence of dental disease, and the following parameters were evaluated for the incisors and the cheek teeth, adapted from Borawski et al., 2024 [19]:

- Malocclusion, which refers to the position of the mandibular and maxillary teeth in relation to each other;
- Retrograde apical elongation of the reserve crowns;
- Overgrowth of the clinical crowns;
- Dental abscesses, defined as a fluid/soft tissue attenuation lesion with peripheral enhancement after intravenous contrast administration;
- Mandibular or maxillary bone deformities secondary to the existence of overgrowth of the clinical crowns or dental abscesses;
- Osteomyelitis secondary to the presence of an abscess of dental origin;
- Inflammatory resorption of the clinical or reserve crowns.

All the above-mentioned elements were classified as present or absent, and unilateral or bilateral involvement, except for malocclusion, retrograde elongation of reserve crowns’ apices and overgrowth of clinical crowns, which were categorized depending on their severity as normal, mildly affected or severely affected. In addition, the head CT examination included evaluation of the sinonasal structures and middle ear cavities for the presence of alterations, including nasal cavity inflammation and otitis media.

Then, the thoracic region was exhaustively reviewed for pulmonary lesions. They were classified as:

- Bronchial: Defined as an increase in bronchial wall thickness involving one or more of the main bronchi, with or without the presence of intraluminal fluid attenuating content.
- Parenchymal: Presence of hyperattenuating lung areas, without lobar volume reduction, and with or without fluid and/or gas attenuating inclusions.
- Pleural: Hyperattenuating areas originating from the pleural region or a significant increase in pleural thickness.

The lesions were also categorized based on their focal or multifocal distribution, the presence or absence of well-defined margins, and the lung lobe involved (left cranial, including both its cranial and caudal segments; left caudal, right cranial, right middle, right caudal or accessory).

In addition, to evaluate the degree of lung expansion and the presence of significant atelectasis, attenuation values were measured using Hounsfield Units (HU) at the level of the accessory lung lobe, where the largest surface area of the lung parenchyma was observed. Three separate 5–10 mm^2^ circular to ovoid regions of interest (ROIs) were manually drawn bilaterally in both the dorsal and ventral aspects of the unaffected lung parenchyma, using only pre-contrast series, and avoiding the broncho-vascular structures. The computer automatically calculated the mean HU values of each ROI, and a total mean value was then obtained for both the dorsal and the ventral compartments. When pulmonary lesions were present, their attenuation values were also measured using the same method.

### 2.4. Statistical Analysis

Descriptive statistics were presented as mean (SD) for continuous variables and counts (percentages) for categorical variables. When stratified by outcome, we display row percentages to facilitate comparison across groups. No formal hypothesis testing was reported in descriptive tables.

Age (years) and sex (female/male) were specified before analysis as the minimal sufficient adjustment set for confounding and were included in the primary multivariable model. Neutered status (intact/neutered) and lop-ear phenotype (no lop/lop) were summarized descriptively and evaluated in univariable screening models; given the limited number of outcome events and the absence of strong univariable associations with pulmonary lesions, they were not retained in the prespecified primary model to preserve parsimony and precision. Balance in these variables was nonetheless examined qualitatively within the sensitivity analyses based on matching and weighting.

For association analyses, we estimated prevalence ratios (PRs) using Poisson regression with a log link and robust (HC0) standard errors, which provides valid large-sample inference and a direct estimate of the PR for a binary outcome. We first fitted univariable Poisson models for each candidate predictor. The primary multivariable model included the composite any-dental-involvement exposure adjusted for age and sex. We reported PRs with 95% confidence intervals (CIs) and two-sided *p*-values, using α = 0.05 without multiplicity adjustment; estimates are interpreted alongside their precision. Two prespecified sensitivity analyses probed robustness to confounding control for the primary exposure. First, we implemented 1:1 nearest-neighbor matching (ATT estimand) using Mahalanobis distance on age with exact matching on sex (treated group defined as any-dental-involvement = Yes). The matched set was analyzed with a Poisson model (log link) adjusted for age and sex, and cluster-robust variance estimators were used with the matched-pair identifier as the clustering unit to account for within-pair correlation. Second, we estimated the Average Treatment effect in the Overlap population (ATO) using overlap weights derived from a logistic propensity score that included age and sex; treated animals received weight 1 − e(X) and controls e(X) so that emphasis was placed on animals with substantial treatment equipoise. We then fit a doubly robust Poisson model (weighted by the overlap weights and adjusted for age and sex) with robust (HC3) variance. This estimand targets the effect among animals in the region of covariate overlap.

### 2.5. Software

Analyses used available cases; no imputation was performed. All computations were carried out in R software (version 4.5.0) with the following packages: tidyverse (version 2.0.0) for data wrangling, rio (version 1.2.3) for data import, gtsummary (version 2.4.0) for descriptive and regression tables, flextable (version 0.9.9) for table export, sandwich (version 3.1.1) and lmtest (version 0.9.40) for robust and cluster-robust inference, MatchIt (version 4.7.2) for matching, WeightIt (version 1.5.0) for propensity-score weighting including overlap weights, and cobalt (version 4.6.1) for covariate-balance diagnostics.

## 3. Results

### Study Population

Among a total of 73 analyzed rabbits, 21 (29%) showed pulmonary lesions, the mean (SD) age was 4.96 (±2.69) years; 34% were female and 51% were neutered. Lop-ear phenotype was present in 36%. Dental disease was identified in 81% of rabbits, reflecting contributions from malocclusion, retrograde elongation, and clinical-crown overgrowth. Among rabbits affected by dental disease, pulmonary lesions were detected in 29%, compared with 14% of rabbits without dental disease. Full distributions by dental subcomponents (severity and laterality) and maxillofacial pathology are reported in Table 1. Regarding comorbidities, sinonasal disease was concurrently identified in 26 of 73 rabbits (36%), while otitis media was present in 29 animals (40%). Within rabbits affected by dental disease (59/73), sinonasal disease and otitis media were identified in 26 (44%) and 29 (49%) cases, respectively. Descriptive analysis did not reveal a consistent association between dental disease severity and the presence of pulmonary lesions.

**Figure 1:**
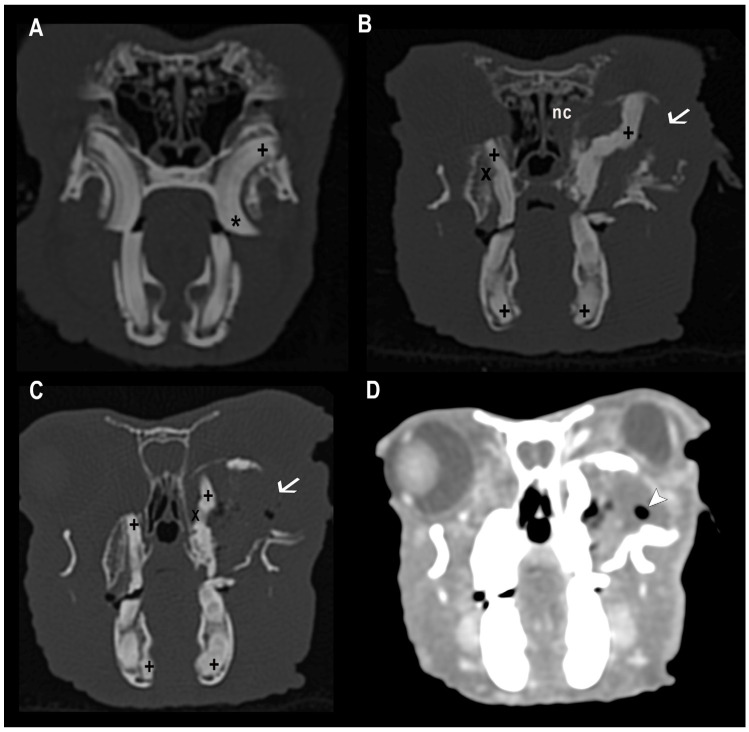
CT transverse images in bone (**A**–**C**) and soft-tissue windows (**D**). (**A**) Mild to moderate dental disease. Bilateral cheek teeth malocclusion with sclerosis of the dental apices. The maxillary cheek teeth show clinical-crown overgrowth (*) and retrograde elongation (+), resulting in secondary cortical thinning of the maxillary bone, more pronounced on the left side. (**B**) Severe dental disease. Bilateral cheek teeth malocclusion, apical sclerosis (+), and inflammatory resorption (X). Elongation of the left maxillary cheek tooth apex causes a periapical abscess with secondary osteolysis and deformation of the maxillary bone (arrow), with extension into the right nasal cavity (nc). (**C**) Severe dental disease. Bilateral cheek teeth malocclusion, apical sclerosis (+), and inflammatory resorption (X). Elongation of the left maxillary cheek tooth apex causes a periapical abscess with secondary osteolysis and deformation of the maxillary bone (arrow). (**D**) Soft-tissue window corresponding to image (**C**). A large, well-defined, fluid-attenuation lesion with peripheral contrast enhancement in the left maxillary bone, consistent with a periapical abscess, note the gas attenuation bubbles within the lesion (white arrowhead).

**Figure 2:**
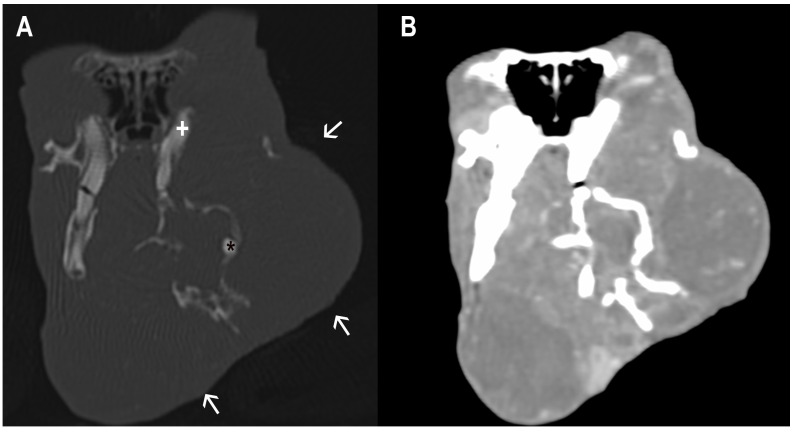
CT transverse images in bone and soft-tissue windows of a rabbit with severe dental disease. The left maxillary and mandibular bones are expanded with multifocal areas of osteolysis (arrows). A thinner, sclerotic left maxillary cheek tooth (+) and a sclerotic fragment of a mandibular cheek tooth (*) are visible (**A**). Multiple, round, well-defined fluid-attenuation lesions with peripheral contrast enhancement, compatible with abscesses, involving both the left maxillary and mandibular regions (**B**).

Among the population of the study, around 81% of the rabbits showed dental disease. Within this group, the most frequent abnormalities included malocclusion of cheek teeth (98%), malocclusion of incisors (31%), apical (retrograde) elongation of the reserve crowns (78%), clinical-crown overgrowth (73%), osteomyelitis (49%), dental abscesses (49%), inflammatory resorption (42%), and maxillary bone deformities (41%) (Figure 1 and Figure 2).

Although this association did not reach statistical significance, the findings were considered relevant. Computed tomography proved to be an effective imaging modality for the detection of both dental and thoracic lesions in rabbits. Pulmonary alterations were identified on CT in 32% of rabbits with dental disease, compared with 14% of rabbits without dental disease. This supports the recommendation that, when CT is performed for dental evaluation, extension of the scan to include the thorax may be clinically useful. However, the coexistence of dental and pulmonary lesions should not be interpreted as evidence of a direct causal relationship. Due to the limited number of cases, only descriptive statistics were applied to characterize the pulmonary lesions. Most of them predominantly affected the lung parenchyma (68.4%), while bronchial and pleural involvement were less common, appearing in 10.5% and 5.3% of the cases, respectively. Indeed, 15% of the rabbits presenting dental disease showed both parenchymal and bronchial lesions. When present, parenchymal lesions were predominantly multifocal in distribution (62.5%), exhibited ill-defined margins (62.5%) and ranged from nodular areas to consolidated lung lobes.

**Figure 3:**
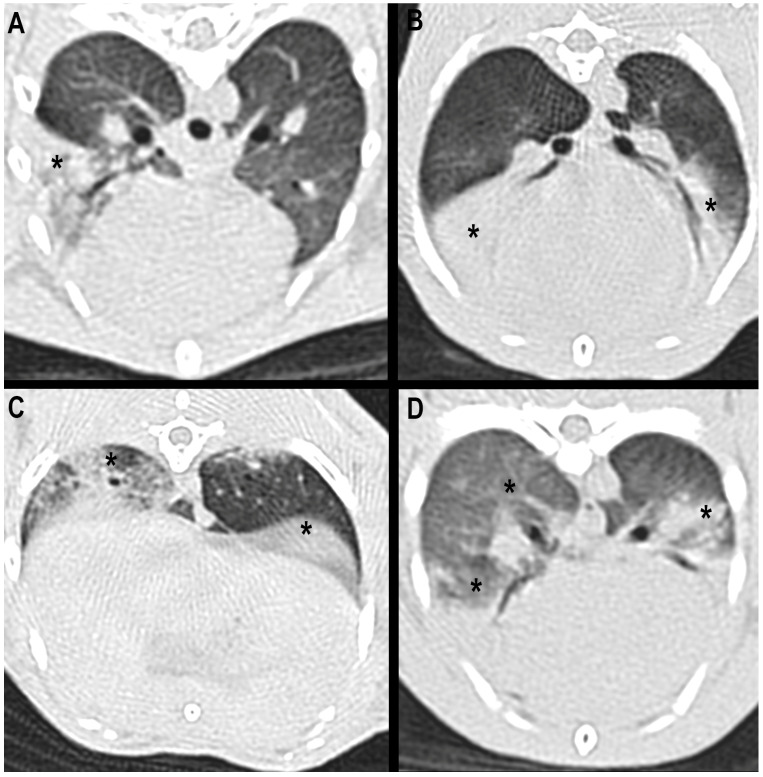
CT transverse images of the thorax in lung window. (**A**–**D**) Soft-tissue attenuation, well-defined lesions (*) involving: (**A**) the right middle lung lobe, (**B**) the right middle and left cranial lung lobes (*), without reduction in the lobar volume, suggestive of lobar consolidation. (**C**,**D**) Multifocally distributed, patchy, ill-defined lesions affecting multiple lung lobes (right and left caudal lung lobes in (**C**), and right middle, right caudal, left cranial and left caudal in (**D**)).

Computed tomographic evaluation revealed that the lesions were bilateral in 56.9% of the animals, more frequently affecting the right hemithorax (66.7%), than the left one (33.3%). Regarding the lobar distribution of the lesions, no major differences were found among the affected lung lobes. The right cranial lung lobe was the one that was most frequently involved, accounting for 25.6% of the patients with dental disease, followed by the right caudal and the left cranial lung lobes, whose values were 23.1% and 20.5%, respectively. The right middle lung lobe exhibited lesions in 18% of the rabbits, and the left caudal one in 12.8% of them. Thus, pulmonary disorders affected the cranio-ventral lung lobes in nearly half of the patients; nevertheless, lesions were found affecting all lung lobes, probably because of their predominantly multifocal nature (Figure 3).

Finally, regarding the Hounsfield Units (HU) measurements, the results demonstrated that in healthy areas of the lungs, the values were lower in the dorsal aspect of the lung (mean [SD] −714.5 [77.4] HU), compared with those obtained in the ventral aspect (mean [SD] −586.4 [95.8] HU). However, when HU were evaluated at the level of the pulmonary lesions, the values markedly increased, reaching a mean value of −132.4 [166.7] HU (range −500 to 76 HU).

Between groups (no pulmonary lesions *n* = 52; pulmonary lesions *n* = 21), age was similar (mean [SD] 4.86 [2.79] vs. 5.21 [2.48] years). Inter-group distributions of sex, neuter status, and lop-ear phenotype were broadly comparable. When examined descriptively by row percentages, pulmonary lesions occurred in 14% of rabbits without dental involvement versus 32% with any dental involvement, suggesting higher crude prevalence among dentally affected animals. No significant association was observed for sinonasal disease or otitis media in relation to pulmonary lesions. Rabbits with sinonasal disease had a crude prevalence ratio of 1.36 (95% CI: 0.55–3.20; *p* = 0.490), while those with otitis media had a prevalence ratio of 1.14 (95% CI: 0.46–2.69; *p* = 0.769), values that do not indicate a meaningful increase in risk. Results are shown in Table 2. In univariable models, across individual dental characteristics (Table 3), none of the univariable PRs showed statistically significant associations with pulmonary lesions; estimates were imprecise, and several categories exhibited sparse data (e.g., unilateral malocclusion).

In the multivariable Poisson model (log link) adjusted for age and sex, any dental involvement was not statistically significantly associated with pulmonary lesions: PR 2.14 (95% CI 0.55–8.38; *p* = 0.275). Age (PR 1.03; 95% CI 0.91–1.17; *p* = 0.654) and sex (female vs. male: PR 1.15; 95% CI 0.53–2.52; *p* = 0.720) were also not significantly associated. Confidence intervals were wide, indicating substantial imprecision (Table 3).

In both sensitivity analyses (Table 4 and Table 5), results were directionally consistent but remained imprecise. Overall, across unadjusted, adjusted, matched, and weighted approaches, we observed no statistically significant association between dental involvement and pulmonary lesions; estimates consistently suggested a possible positive relationship but with wide confidence intervals compatible with no effect.

In the matched analytic sample (no pulmonary lesions *n* = 21; pulmonary lesions *n* = 7), the doubly adjusted model with cluster-robust variance by matched pair yielded PR 3.24 (95% CI 0.65–16.1; *p* = 0.152) for any dental involvement. Estimates remained directionally positive but imprecise.

Using overlap weighting with adjustment for age and sex, any dental involvement again showed no statistically significant association: PR 2.08 (95% CI 0.42–10.4; *p* = 0.371). These results are directionally consistent with the primary model but remain limited by wide confidence intervals.

## 4. Discussion

This study investigated the potential association between dental disease and pulmonary abnormalities in domestic rabbits using computed tomography (CT). This investigation was motivated by necropsy observations in pet rabbits in which severe dental pathology co-occurred with pulmonary lesions, suggesting the hypothesis that such associations might also be detectable in vivo. In human medicine, the association between oral health and respiratory disease is well documented. Several mechanisms have been proposed, including aspiration of periodontal pathogens into the lower respiratory tract, potentially contributing to the development of bacterial pneumonia, chronic bronchitis, and chronic obstructive pulmonary disease [20,21,22]. Oral infections may facilitate colonization of the oropharynx by respiratory pathogens, which are then aspirated into the lungs, especially in individuals with impaired clearance mechanisms [23]. The “oral–lung axis” model suggests that chronic microaspiration of oral microorganisms plays a central role in shaping the lung microbiome, even in healthy individuals [16]. Furthermore, elevated levels of IL-6, TNF-α, and matrix metalloproteinases linked to periodontitis have been implicated in lung tissue inflammation and remodeling [16,24]. In veterinary medicine, an association between oral and respiratory health has also been reported in dogs. The oral cavity may serve as a direct reservoir for bacterial contamination of the lungs, potentially leading to the development of bacterial pneumonia [25]. DeBowes et al. [25] reported that dogs with periodontal disease frequently exhibited histologic evidence of chronic pulmonary changes, including bronchitis, bronchiolitis, and interstitial pneumonia, suggesting a possible association between oral and lower airway pathology. However, in their multiple regression analysis, the periodontal disease score was not a statistically significant predictor for specific lung lesions such as peribronchitis/bronchiolitis (*p* = 0.4445), perivasculitis/alveolitis (*p* = 0.8556), or pneumonia (*p* = 0.9433). Similarly to previous findings in dogs, the study presented here did not identify statistically significant associations between dental disease and pulmonary abnormalities in rabbits; however, descriptive data suggested a higher crude prevalence of pulmonary lesions among rabbits with dental involvement. Based on the authors’ clinical experience, rabbits with severe and chronic dental disease, particularly when accompanied by immunosuppression, may be more susceptible to concurrent pulmonary pathology. Possible mechanisms include bacterial aspiration or hematogenous dissemination; however, these pathways remain speculative and cannot be confirmed within the scope of the present study.

Indeed, the pulmonary abnormalities described in the results section could, to some extent, support these theories, since both bacterial pneumonia and aspiration pneumonia have been associated with lesions in the cranioventral region of the thorax in dogs and cats [26]. Aspiration pneumonia in dogs is usually found to involve one to three lung lobes, with the left and right cranial and right middle lung lobes being the most commonly affected ones, as reported by Constantinescu et al. [27]. To the authors’ knowledge, no similar studies were previously published in rabbits. The present study showed that almost half of the rabbits affected with dental disease exhibited lesions in both cranial lung lobes, a proportion that could rise even higher if lesions identified in the right medial lung lobe were also included, accounting for a total percentage of 64.1% of cases. Thus, the presence of aspiration pneumonia secondary to dental disease could be a plausible explanation for these findings, although other forms of bacterial pneumonia cannot be completely ruled out. Likewise, when lungs are colonized by bacteria from the bloodstream, multifocal ill-defined to nodular lesions are expected, which are not usually confined to the cranioventral regions. Consequently, these findings could correspond with the prevalence of multifocal and ill-defined lesions described in the present study [26,28].

The prevalence of pulmonary lesions in the rabbit in the present study was higher among dentally affected animals (32% vs. 14%); however, in the multivariable Poisson model adjusted for age and sex, dental involvement was not significantly associated with pulmonary lesions (PR 2.14; 95% CI 0.55–8.38; *p* = 0.275), and neither age nor sex showed significant effects. Across unadjusted, adjusted, and sensitivity models, prevalence ratios consistently indicated a possible positive trend (PR range: 2.08–3.24), but wide confidence intervals reflected substantial imprecision. The current data are insufficient to confirm a causal relationship. From a clinical perspective, chronic dental disease in rabbits should be regarded as a systemic condition rather than a localized disorder. Persistent oral pain, reduced food intake, and gastrointestinal dysfunction may contribute to chronic stress, altered nutritional status, and varying degrees of immunosuppression [5]. In this context, the coexistence of dental and pulmonary lesions observed in some rabbits may reflect an increased susceptibility to respiratory disease. These findings highlight the importance of a holistic clinical approach, in which rabbits presenting with dental disease are also evaluated for concurrent thoracic abnormalities, even when the underlying mechanisms remain uncertain. In agreement with Mikoni et al. [10] and the study presented here, age, sex, and reproductive status did not significantly influence the severity or prevalence of respiratory lesions, suggesting that these demographic variables play a limited role in the expression of respiratory pathology in rabbits. Similarly, the lop-eared phenotype did not show a significant association with pulmonary lesions in the current study, which aligns with the lack of effect on rhinitis severity previously reported. However, Mikoni et al. [10] found that non-lop-eared rabbits had significantly higher odds of maxillary sinusitis (proportional OR 6.7; 95% CI, 1.5–31.4; *p* = 0.015). The mean age of the rabbits in the present study (5.21 ± 2.48 years) was slightly lower than that reported in their study (6 years) [10], which may partly explain the lower frequency or reduced severity of respiratory lesions observed. In addition, sinonasal disease was documented in 36% of dentally affected rabbits in our study, clearly lower than the prevalence of rhinitis (71%) and sinusitis (72.5%) identified in that population of rabbits presented primarily for upper respiratory disease [10]. This discrepancy likely reflects differences in the primary clinical indications prompting CT imaging. When compared with other CT-based studies focusing on secondary consequences of dental disease, the prevalence of sinonasal disease in our research (36%) was higher than the 20% reported by Borawski et al. [19], and otitis media was substantially more frequent (40% vs. 6.7%). This variation could be due to differences in the characteristics of the populations examined.

Computed tomography (CT) proved to be a fundamental diagnostic tool, allowing for the simultaneous and detailed evaluation of both dental and pulmonary structures and revealing concurrent alterations in several cases. Despite the absence of statistical significance, CT imaging still revealed patterns compatible with pulmonary extension or secondary consequences of dental disease in selected individuals of the present study. The complex craniofacial consequences of acquired dental disease in rabbits have been documented using CT [20,21]. While the present cross-sectional study included both healthy rabbits and those affected by dental disease, comparison with previous CT-based investigations of dental pathology is most appropriate when restricted to the subgroup of rabbits with dental disease (*n* = 59; 81%). Within this group, the most frequent abnormalities included malocclusion of cheek teeth (98%), malocclusion of incisors (31%), apical (retrograde) elongation of the reserve crowns (78%), clinical-crown overgrowth (73%), osteomyelitis (49%), dental abscesses (49%), inflammatory resorption (42%), and maxillary bone deformities (41%). These findings are broadly consistent with previous CT-based studies [19,29]. Artiles et al. (2020) [29] reported apical elongation in 99% of cases, malocclusion in 100% (with incisor involvement in 28%), tooth resorption or cortical lysis in approximately 55%, periapical lucency with alveolar expansion in 61%, and mandibular canal deformation in 82%. Similarly, Borawski et al. (2024) [19] described malocclusion in 93% of cheek teeth and 63% of incisors, apical elongation in 43%, clinical-crown overgrowth in 43%, dental abscesses in 63%, osteomyelitis in 79% of abscessed rabbits, and inflammatory resorption in 40%. In the present study, the estimated prevalence, particularly for apical elongation, osteomyelitis, inflammatory resorption, and dental abscesses, falls within the ranges reported in previous studies [19,29], supporting the reproducibility of CT findings in chronic dentoalveolar pathology. Collectively, these results reinforce the diagnostic value of CT in characterizing both direct and secondary manifestations of dental disease in rabbits, highlighting its ability to detect osseous remodeling and inflammatory changes that may remain clinically unapparent.

Demographically, the subgroup of dentally affected rabbits in the present study (*n* = 59) showed features comparable to those described in previous CT-based investigations [19,29]. The mean age was 5.14 years, similar to the 5.16 years reported by Artiles et al. [29], and within the range of 3 to 9 years described by Borawski et al. [19] (*n* = 90). Regarding sex distribution, 37 of 59 rabbits (63%) were males and 22 (37%) were females, which mirrors the mild male predominance reported by Artiles et al. (62% males) [29], while Borawski et al. [19] did not provide information on sex distribution. The proportion of lop-eared rabbits in the present study (19/59; 32%) was higher than that reported by Artiles et al. (17%) [29], whereas Borawski et al. [19] did not specify breed distribution. This higher representation of lop-eared rabbits is likely related to the current demographic trend in the pet rabbit population, where lop breeds have become increasingly popular. Nevertheless, all investigations consistently identify adult, middle-aged rabbits as the group most frequently affected by acquired dental disease. No statistically significant association between age, sex, lop-eared breed, and rabbits with dental and pulmonary disease was observed in the study presented here.

From a clinical perspective, CT has proven essential in detecting subclinical respiratory involvement, even in patients without obvious respiratory symptoms; in fact, it is considered the gold standard technique for the evaluation of lung diseases in rabbits [30]. This supports its role as a recommended screening modality in individuals with advanced dental pathology. A recently published large-scale study identified pulmonary emphysema as a relatively frequent and progressive finding in rabbits, predominantly affecting the cranial lung lobes and occasionally leading to complications such as rib fractures or secondary pneumothorax [31]. The absence of both emphysema and pneumothorax in the present population may reflect the younger average age of the study group, the exclusion of severely dyspneic individuals, or hospital case-selection bias. This finding is consistent with previous reports describing spontaneous pneumothorax as a rare but severe condition in pet rabbits, occasionally associated with emphysematous bullae, cavitary lesions, or neoplasia [32]. Nevertheless, both studies highlight the importance of thoracic CT for detecting incidental or early respiratory alterations that may remain clinically silent in rabbits.

This study had several limitations. The reduced sample size and the cross-sectional nature of the study may limit the ability to detect statistically significant differences and to confirm a causal relationship between dental and pulmonary disorders. In addition, the absence of microbiological analysis and histological confirmation could further restrict the scope of the study, as subtle pulmonary lesions can sometimes be difficult to differentiate from atelectasis on CT. To minimize these errors, Hounsfield Units were measured in each patient, showing results for sedated patients similar to those published by Sargo et al. [30], with values being slightly higher in the dorsal region (−714.5 [77.4] UH vs. −684.2 [19] UH; mean [SD]) and slightly lower in the ventral region (−586.4 [95.8] UH vs. −661 [35] UH; mean [SD]). Since poorly aerated lung tissue is considered when HU values decrease below −499 HU, no significant atelectasis was observed in the rabbits included in the study. Indeed, the same authors [30] have demonstrated that sedated rabbits show higher mean lung volumes and attenuation values on CT compared to anesthetized ones. Finally, the presence of concurrent conditions involving the middle ear or the nasal cavity, despite not being associated with an increased risk of pulmonary lesions in this study, may still have influenced the findings to some extent regarding pulmonary disease.

Further prospective research, including histopathological confirmation, microbiological analysis, and assessment of systemic inflammatory markers, will be necessary to elucidate the underlying mechanisms and determine whether chronic dental inflammation may contribute to respiratory pathology in this species.

## 5. Conclusions

In conclusion, although this study did not identify a statistically significant association between dental disease and pulmonary abnormalities in domestic rabbits, descriptive data indicated a higher prevalence of pulmonary lesions among animals with dental disease. Computed tomography proved valuable for concurrently assessing dental and thoracic structures, allowing detection of coexisting osseous and pulmonary alterations in several cases. These findings emphasized the value of CT as a comprehensive diagnostic tool in rabbits with dental disease and suggested that local or systemic interactions between oral and respiratory health may exist. Further prospective investigations are warranted to clarify this relationship and to determine whether early diagnosis and management of dental pathology could help prevent respiratory complications in this species.

## Figures and Tables

**Table 1:** Baseline characteristics of the study group.

Variables	*N* = 73 ^1^
Pulmonary lesions	
No	52 (71%)
Yes	21 (29%)
Lop	
No	47 (64%)
Yes	26 (36%)
Age	
Mean (SD)	4.96 (2.69)
Sex	
Male	48 (66%)
Female	25 (34%)
Neutered	
No	36 (49%)
Yes	37 (51%)
Dental involvement	
No	14 (19%)
Yes	59 (81%)
Malocclusion—Incisors	
Normal	50 (68%)
Mild	9 (12%)
Severe	14 (19%)
Malocclusion—Cheek teeth	
Normal	15 (21%)
Mild	31 (42%)
Severe	27 (37%)
Malocclusion	
Normal	15 (21%)
Unilateral	6 (8.2%)
Bilateral	52 (71%)
Retrograde elongation—Incisors	
Normal	48 (66%)
Mild	11 (15%)
Severe	14 (19%)
Retrograde elongation—Cheek teeth	
Normal	27 (37%)
Mild	23 (32%)
Severe	23 (32%)
Retrograde elongation	
Normal	27 (37%)
Unilateral	12 (16%)
Bilateral	34 (47%)
Clinical-crown overgrowth—Incisors	
Normal	62 (85%)
Mild	9 (12%)
Severe	2 (2.7%)
Clinical-crown overgrowth—Cheek teeth	
Normal	24 (33%)
Mild	38 (52%)
Severe	11 (15%)
Clinical-crown overgrowth	
Normal	23 (32%)
Unilateral	7 (9.6%)
Bilateral	43 (59%)
Maxillary bone deformities	
No	49 (67%)
Yes	24 (33%)
Mandibular bone deformities	
No	37 (51%)
Yes	36 (49%)
Dental abscesses	
No	44 (60%)
Yes	29 (40%)
Osteomyelitis	
No	30 (51%)
Yes	29 (49%)
Inflammatory resorption	
No	34 (58%)
Yes	25 (42%)
Sinonasal disease	
No	47 (64%)
Yes	26 (36%)
Otitis media	
No	44 (60%)
Yes	29 (40%)

^1^ Data are presented as *n* (%) unless otherwise indicated; continuous variables are presented as mean (SD).

**Table 2:** Univariable associations with pulmonary lesions.

	Descriptive		Univariable PR	
Variables	No Pulmonary Lesions *N* = 52 ^1^	Pulmonary Lesions *N* = 21 ^1^	PR (95% CI)	*p*-Value
Lop				
No	35 (74%)	12 (26%)	—	
Yes	17 (65%)	9 (35%)	1.36 (0.55–3.20)	0.490
Age			1.04 (0.88–1.21)	0.662
Mean (SD)	4.86 (2.79)	5.21 (2.48)		
Sex				
Male	35 (73%)	13 (27%)	—	
Female	17 (68%)	8 (32%)	1.18 (0.47–2.80)	0.710
Neutered				
No	26 (72%)	10 (28%)	—	
Yes	26 (70%)	11 (30%)	1.07 (0.45–2.57)	0.876
Malocclusion—Incisors				
Normal	36 (72%)	14 (28%)	—	
Mild	7 (78%)	2 (22%)	0.79 (0.12–2.84)	0.760
Severe	9 (64%)	5 (36%)	1.28 (0.41–3.33)	0.640
Malocclusion—Cheek teeth				
Normal	12 (80%)	3 (20%)	—	
Mild	21 (68%)	10 (32%)	1.61 (0.49–7.19)	0.468
Severe	19 (70%)	8 (30%)	1.48 (0.43–6.76)	0.561
Malocclusion				
Normal	12 (80%)	3 (20%)	—	
Unilateral	6 (100%)	0 (0%)	0.00 (0.00–Inf)	0.994
Bilateral	34 (65%)	18 (35%)	1.73 (0.51–5.88)	0.379
Retrograde elongation of reserve crowns’ apices—Incisors				
Normal	33 (69%)	15 (31%)	—	
Mild	8 (73%)	3 (27%)	0.87 (0.20–2.64)	0.830
Severe	11 (79%)	3 (21%)	0.69 (0.16–2.08)	0.551
Retrograde elongation of reserve crowns’ apices—Cheek teeth				
Normal	20 (74%)	7 (26%)	—	
Mild	14 (61%)	9 (39%)	1.51 (0.56–4.22)	0.414
Severe	18 (78%)	5 (22%)	0.84 (0.25–2.63)	0.764
Retrograde elongation of reserve crowns’ apices				
Normal	20 (74%)	7 (26%)	—	
Unilateral	9 (75%)	3 (25%)	0.96 (0.21–3.47)	0.958
Bilateral	23 (68%)	11 (32%)	1.25 (0.49–3.39)	0.647
Clinical crowns overgrowth—Incisors				
Normal	44 (71%)	18 (29%)	—	
Mild	8 (89%)	1 (11%)	0.38 (0.02–1.85)	0.350
Severe	0 (0%)	2 (100%)	3.44 (0.55–11.9)	0.097
Clinical crowns overgrowth— Cheek teeth				
Normal	18 (75%)	6 (25%)	—	
Mild	27 (71%)	11 (29%)	1.16 (0.44–3.36)	0.773
Severe	7 (64%)	4 (36%)	1.45 (0.37–5.09)	0.562
Clinical crowns overgrowth				
Normal	18 (78%)	5 (22%)	—	
Unilateral	5 (71%)	2 (29%)	1.31 (0.19–6.10)	0.744
Bilateral	29 (67%)	14 (33%)	1.50 (0.57–4.63)	0.438
Maxillary bone deformities				
No	35 (71%)	14 (29%)	—	
Yes	17 (71%)	7 (29%)	1.02 (0.39–2.45)	0.964
Mandibular bone deformities				
No	26 (70%)	11 (30%)	—	
Yes	26 (72%)	10 (28%)	0.93 (0.39–2.22)	0.876
Dental abscesses				
No	30 (68%)	14 (32%)	—	
Yes	22 (76%)	7 (24%)	0.76 (0.29–1.82)	0.551
Osteomyelitis				
No	17 (57%)	13 (43%)	—	
Yes	23 (79%)	6 (21%)	0.48 (0.17–1.21)	0.134
Inflammatory resorption				
No	22 (65%)	12 (35%)	—	
Yes	18 (72%)	7 (28%)	0.79 (0.30–1.97)	0.626
Sinonasal disease				
No	35 (74%)	12 (26%)	—	
Yes	17 (65%)	9 (31%)	1.36 (0.55–3.20)	0.490
Otitis media				
No	32 (73%)	12 (27%)	—	
Yes	20 (69%)	9 (31%)	1.14 (0.46–2.69)	0.769

Abbreviations: CI = Confidence Interval, PR = Prevalence Ratio. ^1^ Data are presented as *n* (%) unless otherwise indicated; continuous variables are presented as mean (SD).

**Table 3:** Primary adjusted analysis of any dental involvement and pulmonary lesions.

	Descriptive		Univariable PR		Multivariable PR	
Variables	No Pulmonary Lesions *N* = 52 ^1^	Pulmonary Lesions *N* = 21 ^1^	PR (95% CI)	*p*-Value	PR (95% CI)	*p*-Value
Dental involvement						
No	12 (86%)	2 (14%)	—		—	
Yes	40 (68%)	19 (32%)	2.25 (0.65–14.2)	0.274	2.14 (0.55–8.38)	0.275
Age			1.04 (0.88–1.21)	0.662	1.03 (0.91–1.17)	0.654
Mean (SD)	4.86 (2.79)	5.21 (2.48)				
Sex						
Male	35 (73%)	13 (27%)	—		—	
Female	17 (68%)	8 (32%)	1.18 (0.47–2.80)	0.710	1.15 (0.53–2.52)	0.720

Abbreviations: CI = Confidence Interval, PR = Prevalence Ratio. ^1^ Data are presented as *n* (%) unless otherwise indicated; continuous variables are presented as mean (SD).

**Table 4:** Sensitivity analysis A: 1:1 matching with cluster-robust inference.

	Descriptive		Multivariable PR	
Variables	No Pulmonary Lesions *N* = 21 ^1^	Pulmonary Lesions *N* = 7 ^1^	PR (95% CI)	*p*-Value
Dental involvement				
No	12 (86%)	2 (14%)	—	
Yes	9 (64%)	5 (36%)	3.24 (0.65–16.1)	0.152
Age			0.87 (0.64–1.18)	0.384
Mean (SD)	5.12 (2.69)	4.71 (1.89)		
Sex				
Female	4 (67%)	2 (33%)	—	
Male	17 (77%)	5 (23%)	0.77 (0.24–2.52)	0.669

Abbreviations: CI = Confidence Interval, PR = Prevalence Ratio. ^1^ Data are presented as *n* (%) unless otherwise indicated; continuous variables are presented as mean (SD).

**Table 5:** Sensitivity analysis B: overlap-weighted doubly robust model.

	Descriptive		Multivariable PR	
Variables	No Pulmonary Lesions *N* = 52 ^1^	Pulmonary Lesions *N* = 21 ^1^	PR (95% CI)	*p*-Value
Dental involvement				
No	12 (86%)	2 (14%)	—	
Yes	40 (68%)	19 (32%)	2.08 (0.42–10.4)	0.371
Age			1.02 (0.75–1.38)	0.922
Mean (SD)	4.86 (2.79)	5.21 (2.48)		
Sex				
Female	17 (68%)	8 (32%)	—	
Male	35 (73%)	13 (27%)	0.64 (0.20–2.08)	0.457

Abbreviations: CI = Confidence Interval, PR = Prevalence Ratio. ^1^ Data are presented as *n* (%) unless otherwise indicated; continuous variables are presented as mean (SD).

## Data Availability

No new data were created or analyzed in this study. Data sharing is not applicable to this article.
